# Effects of dexmedetomidine on postoperative cognitive function of sleep deprivation rats based on changes in inflammatory response

**DOI:** 10.1080/21655979.2021.1981757

**Published:** 2021-10-08

**Authors:** Bin Guo, Chan Chen, Lin Yang, Rong Zhu

**Affiliations:** Department of Anesthesiology, the Second Xiangya Hospital, Central South University, Changsha, Hunan, China

**Keywords:** Sleep deprivation, dexmedetomidine, inflammatory response, cognitive function, rat

## Abstract

We aimed to assess the effects of dexmedetomidine (DEX) on postoperative cognitive function of sleep deprivation (SD) rats based on changes in inflammatory response. Male rats were randomly divided into blank control (C), SD, DEX, and SD+DEX groups. The SD model was established through intraperitoneal injection of DEX. The escape latency was detected through Morris water maze test daily, and the mechanical withdrawal threshold and thermal withdrawal latency were detected for 8 d. The content of malondialdehyde (MDA) and activity of superoxide dismutase (SOD) in hippocampus homogenate were determined, and the morphological changes in neurons were detected through Nissl staining. The concentration of interleukin-1β (IL-1β), tumor necrosis factor-α (TNF-α), and IL-6 in the hippocampus was detected by enzyme-linked immunosorbent assay, and the Rac1/protein kinase B (AKT)/nuclear factor-κB (NF-κB) expressions were detected by Western blotting. The changes in immunofluorescence localization of NF-κB were observed by confocal microscopy. Compared with SD group, the escape latency was shortened, original platform-crossing times increased, MDA content declined, SOD activity rose, neurons were arranged orderly and number of Nissl bodies increased in the hippocampal CA1 region, levels of IL-1β, TNF-α, and IL-6 in the hippocampus decreased, Rac1/AKT/NF-κB expressions were down-regulated, and proportion of NF-κB entering the nucleus declined in SD+DEX group (*P* < 0.05). DEX can effectively alleviate postoperative hippocampal inflammation and improve cognitive function of SD rats. The ability of DEX to relieve oxidative stress of hippocampal neurons, restore damaged cells, and reduce hippocampal inflammation in SD rats may be related to the Rac1/AKT/NF-κB pathway.

## Introduction

Sleep deprivation (SD), which is common among night-shift workers such as medical professionals and locomotive attendants, can lead to anxiety, depressive symptoms, and impaired judgment. SD is one of the main causes for neurobehavioral and cognitive dysfunction [1]. Postoperative cognitive dysfunction (POCD) is often manifested as changes in personality, memory, attention, language comprehension, mental activity, and social activity after surgery. The patients’ own state, anesthesia process, operation method, and sleep quality are closely related to POCD. In particular, the association between sleep insufficiency or poor sleep quality and POCD has attracted increasing attention [2]. Dexmedetomidine (DEX) is a new highly selective α2 adrenergic receptor agonist and an effective sedative for the short-term sedation of mechanically ventilated patients, exerting a potent analgesic effect [3]. Although α2 receptor agonists exert effective neuroprotective effects during and after neurosurgery, the mechanism remains unclear. In addition to hypnotic sedation and analgesia, DEX has an anti-inflammatory effect [4]. In the case of intestinal ischemia, DEX can inhibit the apoptosis and inflammatory response of intestinal mucosal epithelial cells and promote intestinal injury repair. According to *in vivo* and *in vitro* animal experiments, DEX can protect against ischemia-reperfusion injury in the heart, kidneys, and brain [5,6]. Inflammatory response in the hippocampus is closely related to POCD. The hippocampus is the main functional area of learning and memory, and its function can be damaged by massive inflammatory responses, thus harming the cognitive ability. Wuri *et al*. [7] performed partial hepatectomy for rats and found that the learning and memory abilities of rats were weakened after operation, and the expressions of inflammatory factors in the hippocampus were also up-regulated. Therefore, inhibiting the central inflammatory response may be effective for improving postoperative cognitive function. However, its specific anti-inflammatory mechanism has not been verified, and whether preoperative drug administration can effectively improve the postoperative cognition of patients with sleep disorders remains largely unknown. In this study, the SD rat model was successfully established, based on which the mechanism of DEX for postoperative cognitive function and inflammatory response was explored. We hypothesized that DEX can affect the oxidative stress response of hippocampal neurons by regulating the Rac1/AKT/NF-κB signaling pathway, thereby mitigating the cognitive decline caused by sleep deprivation. The findings provide a reference for future clinical research.

## Materials and methods

### Experimental animals, main reagents and apparatus

Male rats [50 days old, (200 ± 2) g] were purchased from Beijing Vital River Laboratory Animal Co., Ltd., and fed in the Laboratory Animal Center of our hospital. This study has been approved by the hospital’s animal ethics committee on March 17th, 2021 (approval no. DW0982335).

DEX injection (NMPN H20110086) was provided by Jiangsu NHWA Pharmaceutical Co., Ltd. Morris water maze was provided by Huaibei Zhenghua Biological Equipment Co., Ltd. Anesthetics were provided by GIBCO. Nissl staining solution was obtained from Shanghai Beyotime Biotechnology Corporation. Enzyme-linked immunosorbent assay (ELISA) kits were bought from Shanghai Senxiong Biotech Co., Ltd. Western blotting kits and antibodies were obtained from Rebstock (Germany).

Bio-Rad gel imaging system was purchased from Bio-Rad. A −80°C cryogenic refrigerator was obtained from WIGGENS (Germany). A Leica RM2135 microtome was bought from Leica (Germany). A high-speed refrigerated centrifuge was purchased from Beijing Liuyi Instrument Factory. A flow cytometer was provided by Thermo (USA).

### *Model establishment and grouping* [8]

After adaptive feeding, the rats were randomly divided into blank control group (C group, n = 20), SD group (n = 30), DEX group (n = 30), and SD + DEX group (n = 30). Except C group fed with double distilled water, the 48-h SD model was established by the platform water environment method [7] in all groups: A 110 cm × 60 cm × 40 cm box was prepared, in which 15 platforms with the height of 8.0 cm and the diameter of 6.5 cm were set. The platforms were separated by 15 cm and filled with water around, which were about 1.0 cm higher than the water surface. The rats were allowed to eat, drink, and move freely on platforms. The room temperature was maintained at 22–24°C. Sleep was deprived from 9:00 am to 9:00 pm for eight consecutive days. For DEX group and C group, the same large platform water environment method was used: The rats were placed in the water tank with a large platform, and they were allowed to eat, drink, and sleep freely on the platform. For DEX group and SD+DEX group, DEX was intraperitoneally injected (30 μg/kg) once a day until the end of Morris water maze (place navigation) test. An equal volume of normal saline was injected in the same way for SD group and C group.

### *Morris water maze test* [9]

Two weeks after operation, the Morris water maze test was performed in two parts: (1) Place navigation test: Before the test, the rats received adaptive swimming training twice in the morning and twice in the afternoon (2 min/time), and then the test was conducted after 1 day. The Morris water maze was a circular water tank equally divided into four quadrants, and a platform was placed at the center of one quadrant. The place navigation training was given for five consecutive days, twice in the morning and twice in the afternoon at an interval of 120 s. Then, the rats facing the wall were placed into water from different quadrants at the same time point, and the duration from entering water to climbing onto the platform (escape latency) was recorded. If the rats failed to find the platform within 60 s, they were guided to the platform for 10 s, and the escape latency was recorded as 60 s. The average of escape latency obtained 4 times was taken as the final latency of the day. (2) Spatial probe test: After place navigation test, the platform was removed. Then the rats facing the wall were placed into water from the same entry point once a day for 2 days, and the original platform-crossing times were recorded within 60 s. The percentage of swimming time within the target quadrant (area where the original platform was located) in total was calculated twice, and the average was taken as the final result.

### *Exploration after laparotomy* [10]

The rats were anesthetized through intraperitoneal injection of 10% chloral hydrate (3 μL/kg), followed by skin preparation and disinfection in a sterile environment. A 2 cm-long median incision was made 1 cm at the front end of the urethral orifice toward the head. The skin, subcutaneous tissues, and peritoneum were cut open layer by layer to open the abdominal cavity. Then, the intestine was taken out of the abdominal cavity, the liver, spleen, kidneys, and bladder were gently explored with cotton swabs, and the abdomen was closed with sutures after 30 min. During operation, the body temperature of rats was kept at about 37°C using heating blankets.

### *Measurement of oxidative stress indices in hippocampal tissues* [11]

After the Morris water maze test, four rats were selected from each group using a random number table, and quickly decapitated. The fresh hippocampus was harvested, homogenized with a homogenizer, and centrifuged at 4°C. Then, the supernatant was collected and detected according to the instructions of ELISA kits. The optical density (OD) at a wavelength of 450 nm was measured using a microplate reader, and the polynomial quadratic regression equation of the standard curve was solved. The OD value was substituted into the standard curve to calculate the activity of superoxide dismutase (SOD) and content of malondialdehyde (MDA).

### *Nissl staining* [12]

After the Morris water maze test, five rats were selected from each group and immediately anesthetized with chloral hydrate, and the chest was cut open to expose the heart. Afterward, 100 mL of normal saline and paraformaldehyde were quickly perfused into the left ventricle for fixation. Then, the rats were sacrificed, and the hippocampus was carefully peeled off, fixed in paraformaldehyde for 4–6 h, dehydrated, embedded in paraffin, and sliced into sections. The sections were stained with Nissl staining solution for 5–10 min, dehydrated with 95% ethanol for 2 min, and transparentized with xylene for 5 min. Finally, the morphological changes in neurons in the hippocampal CA1 region were observed under an optical microscope.

### *Detection of tumor necrosis factor-α (TNF-α), interleukin-1β (IL-1β), and IL-6 levels by ELISA* [13]

The levels of IL-1β, TNF-α, and IL-6 in the hippocampus were detected by ELISA. The cryopreserved hippocampus samples were added with an appropriate amount of normal saline, fully homogenized for 10 min and centrifuged, and the supernatant was harvested. The kits were taken from the refrigerator and equilibrated to room temperature, and the test strips were taken out. Standard and sample wells were set and added with 50 μL of standards and samples, respectively. Subsequently, 100 μL of horseradish peroxidase-labeled antibodies were added into each well, and the wells were sealed with sealing tape, followed by incubation at 37°C for 60 min. Then, the wells were opened, the liquid in each well was patted dry, the wells were filled with washing solution and placed for 1 min, and then the liquid in each well was spin-dried again (5 times). Afterward, 50 μL of substrate was added into each well in dark and incubated at 37°C for 20 min. Then, 50 μL of stop buffer was added into each well, and the OD value of each well at 450 nm was measured within 15 min. The standard regression curve was plotted, with the standard concentration as the abscissa, and the corresponding OD value as the ordinate, based on which the sample concentration was read.

### *Detection of protein expressions by Western blotting* [14]

After anesthesia with intraperitoneal injection of 10% chloral hydrate (3 μL/kg), the spinal cord was transected, the brain was quickly harvested, and bilateral hippocampi were separated on ice. The hippocampus was stored in a refrigerator at −80°C. The samples in each group were homogenized, from which total protein was extracted using kits. Then, the protein was quantified by BCA (bicinchoninic acid) according to the instructions. After the gel for sodium dodecyl sulfate-polyacrylamide gel electrophoresis was prepared, the protein was subjected to electrophoresis, transferred onto a membrane, sealed, and incubated with primary antibodies [Rac1 (1:2000 diluted, Abcam, UK), AKT (1:500 diluted, Abcam, UK) and NF-κB (1:800 diluted, Abcam, UK)] and secondary antibody [horseradish peroxidase-labeled goat anti-rat IgG (1:800 diluted, Abcam, UK)]. Finally, the protein expression level was analyzed through exposure and scanning using image analysis software.

### *Observation of immunofluorescence location of nuclear factor-κB (NF-κB) by confocal microscopy* [15]

After dissection, the hippocampus was harvested, placed into a penicillin bottle containing Hanks solution, rinsed with Hanks solution, roughly cut, rinsed with Hanks solution again, finely cut, prepared into single cell suspension and inoculated, followed by subculture till the logarithmic growth phase. The cells in the logarithmic growth phase were inoculated into a slide confocal small dish (9 × 10^4^ cells/mL), and incubated in an incubator with 5% CO_2_ at 37°C overnight. For SD+DEX group and DEX group, DEX at a final concentration of 2.0 μg/kg was added. For C group and SD group, the same amount of 0.9% sodium chloride injection was added. After 0.5 h, the cells were fixed with fixative for 0.5 h, permeabilized with Triton X-100 for 0.5 h, sealed with serum blocking buffer for 0.5 h, and incubated with primary antibodies (1:50) and secondary antibody (1:500) for 1 h, followed by nuclear staining with 4’,6-diamidino-2-phenylindole (1:2000) and mounting. Finally, the sections were observed under a confocal microscope, and the results were analyzed.

### Statistical analysis

SPSS 25.0 software was used for statistical analysis. All data were tested for homogeneity of variance and normal distribution. The measurement data conforming to normal distribution were represented as mean ± standard deviation. The comparisons between two groups were performed by the t test, and those among multiple groups were carried out by one-way analysis of variance. Graphpad 5.0 was used for plotting. P < 0.05 was considered statistically significant.

## Results

### Effects of DEX on MDA level and SOD activity in hippocampal tissues of SD rats

MDA is the final product of lipid peroxidation caused by the damage of unsaturated fatty acids in biomembranes by oxygen free radicals. The levels of MDA in serum and tissues represent the speed and intensity of lipid peroxidation and indirectly reflect the degree of free radical damage, as a crucial index of cerebral ischemic damage [16]. SOD is a key enzyme for scavenging superoxide anions, with its activity indirectly reflecting the ability to scavenge oxygen free radicals [17]. Therefore, combining the two indices reflects the production and elimination of free radicals. It has previously been reported that sleep deprivation caused changes in SOD and MDA levels [18].

**Figure 1.**
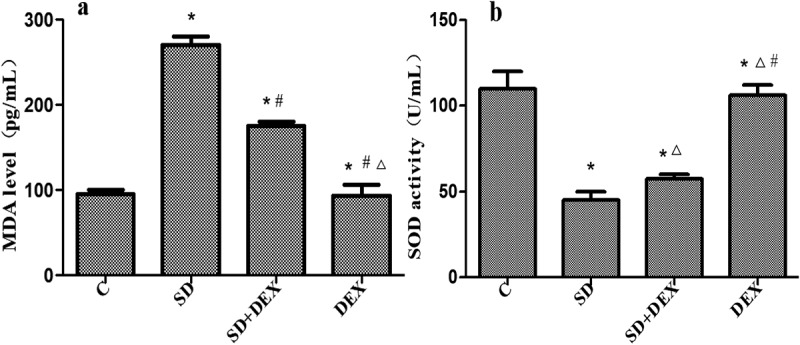
Oxidative stress indices. *P < 0.05 *vs*. C group; #P < 0.05, ∆P < 0.01 *vs*. SD group

The content of MDA in the hippocampus was significantly higher, and the activity of SOD was significantly lower in SD group than those in C group (P < 0.05). The content of MDA in the hippocampus was significantly lower, and the activity of SOD was significantly higher in SD + DEX group than those in SD group (P < 0.05). The MDA content and SOD activity in the hippocampus of DEX group had significant differences from those of SD group or SD + DEX group (P < 0.01) (Figure 1).

### Postoperative Morris water maze test results

The Morris water maze test forces experimental animals to swim and learn to find a platform hidden in water to test their learning and memory abilities for spatial position and orientation. The cognitive function of rats was evaluated by this test herein [19].

**Figure 2.**
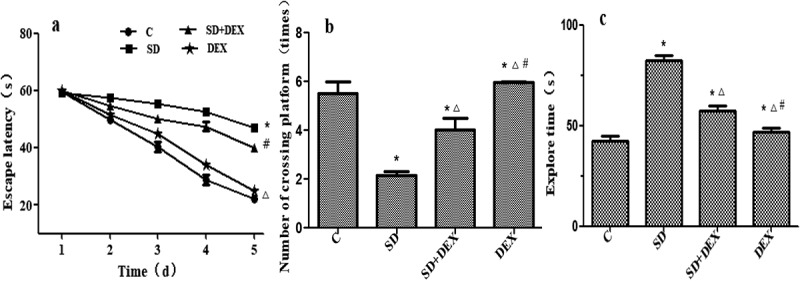
Postoperative Morris water maze test results. (a) Escape latency; (b) platform-crossing times; and (c) probe time. *P < 0.05 *vs*. C group; #*P* < 0.05, ∆*P* < 0.01 *vs*. SD group

Compared with C group, the escape latency in place navigation test was significantly prolonged, the original platform-crossing times in spatial probe test reduced, and the probe time increased in SD group (*P* < 0.05). Compared with SD group, the escape latency in place navigation test was significantly shortened, the original platform-crossing times in spatial probe test increased, and the probe time decreased in SD + DEX group (*P* < 0.05) (Figure 2).

### Nissl staining results of neurons in the hippocampal CA1 region

The hippocampus, especially hippocampal CA1, plays essential roles in participating in the learning process and memory storage [20].

**Figure 3.**
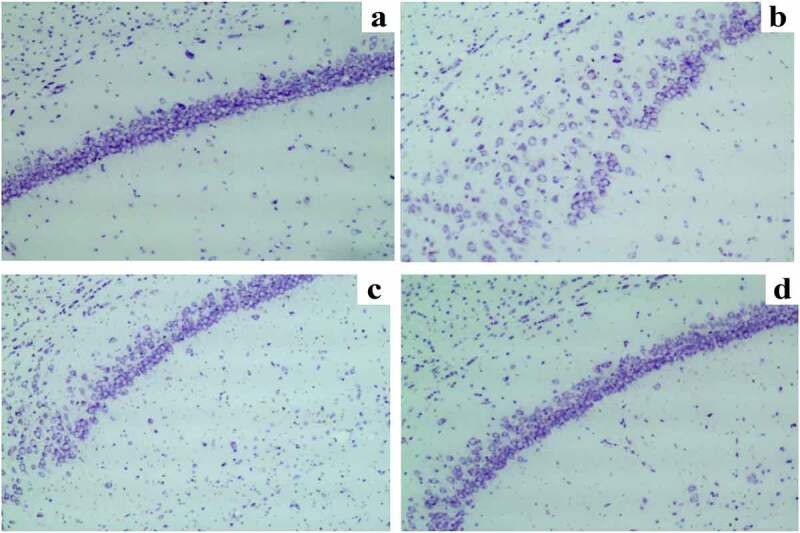
Nissl staining results of neurons in the hippocampal CA1 region. (a) C group; (b) SD group; (c) SD + DEX group; and (d) DEX group

It was observed through Nissl staining that the neuronal damage was obvious, the number of Nissl bodies reduced, and the hippocampal neurons were arranged disorderly with karyopyknosis in the hippocampal CA1 region in SD group compared with those in C group. The neuronal damage was obviously relieved, the number of Nissl bodies increased, the neurons were arranged orderly and distributed uniformly, and karyopyknosis was alleviated in the hippocampal CA1 region in SD + DEX group compared with those in SD group. The Nissl staining results showed that in DEX group, the neuronal damage was mitigated more remarkably than that in SD group or SD + DEX group (Figure 3).

### Expressions of TNF-α, IL-1β, and IL-6 in the hippocampus

Inflammatory cytokines, such as TNF-α, IL-1β, and IL-6, have regulatory effects on many physiological activities of the central nervous system and widely participate in the pathophysiological processes of related diseases [21].

**Figure 4.**
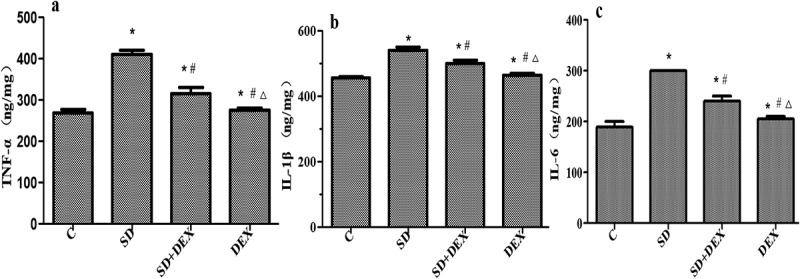
Expressions of TNF-α, IL-1β, and IL-6 in the hippocampus. *P < 0.05 *vs*. C group; #*P* < 0.05, ∆*P* < 0.01 *vs*. SD group

The levels of IL-1β, TNF-α, and IL-6 in the hippocampus significantly increased in SD group compared with those in C group, while they decreased in SD+DEX group compared with those in SD group (*P* < 0.05). The above indices were more significantly different in DEX group (*P* < 0.01) (Figure 4).

### Protein expressions of Rac1/AKT/NF-κB in the hippocampus

The secretion of inflammatory cytokines TNF-α, IL-1β and IL-6 is regulated by the Rac1/AKT/NF-κB signaling pathway. The activated NF-κB is translocated from the cytoplasm to the nucleus, then binding target genes and promoting the secretion of TNF-α, IL-1β, and IL-6 [22].

**Figure 5.**
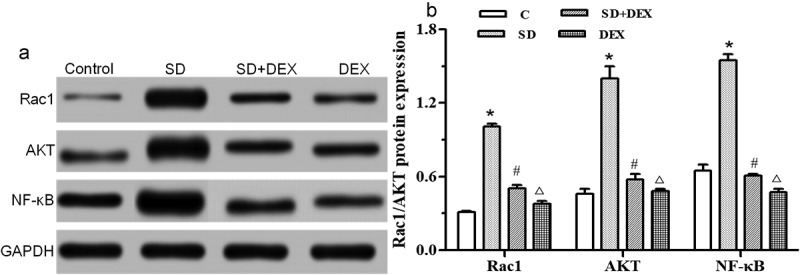
Protein expressions of Rac1/AKT/NF-κB in the hippocampus. (a) Protein expression detected by Western blotting; (b) relative protein expression. *P < 0.05 *vs*. C group; #*P* < 0.05, ∆*P* < 0.01 *vs*. SD group. GAPDH: Glyceraldehyde 3-phosphate dehydrogenase

The Rac1/protein kinase B (AKT)/NF-κB expressions in the hippocampus were detected by Western blotting. The protein expressions of Rac1, AKT, and NF-κB were significantly higher in SD group than those in C group (*P* < 0.05). The protein expressions of Rac1, AKT, and NF-κB were significantly lower in SD+DEX group than those in SD group (*P* < 0.05), and their levels declined in DEX group (*P* < 0.01) (Figure 5).

### Immunofluorescence localization of NF-κB in the hippocampus

**Figure 6.**
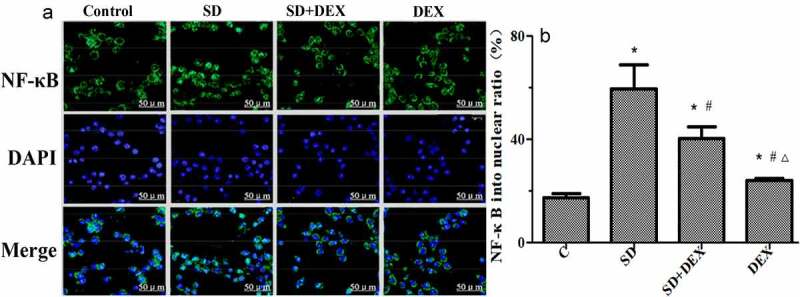
Immunofluorescence localization of NF-κB in the hippocampus. (a) Immunofluorescence localization of NF-κB; (b) proportion of NF-κB entering the nucleus (%). *P < 0.05 *vs*. C group; #*P* < 0.05, ∆*P* < 0.01 *vs*. SD group. DAPI: 4’,6-Diamidino-2-phenylindole

Confocal microscopy revealed that NF-κB was significantly activated, and the proportion of NF-κB entering the nucleus rose after high-tidal-volume mechanical ventilation in SD group compared with that in C group (*P* < 0.05). In SD + DEX group and DEX group, the entry of NF-κB into the nucleus in the hippocampus was significantly inhibited, and the proportion of NF-κB entering the nucleus declined (*P* < 0.05 or *P* < 0.01) (Figure 6).

## Discussion

Sleep is a complete rest state required to eliminate fatigue, and sleep disorders affect the functional recovery. In recent years, the association between sleep insufficiency or poor sleep quality and POCD has attracted increasing attention. DEX can improve the sleep structure and prolong the sleep time of mechanically ventilated patients, thereby raising the sleep quality. Moreover, DEX can effectively relieve pain and reduce the postoperative incidence rates of adverse reactions and cognitive dysfunction in elderly patients undergoing laparoscopic treatment, thereby greatly improving postoperative cognitive function [23]. In this study, SD rats had declined learning and memory functions, which were ameliorated after DEX intervention. The potential protective effect of DEX on brain injury may be related to its ability to resist oxidative stress damage in brain tissues [24]. SOD and MDA are markers for oxidative stress. It is speculated that DEX exerts a neuroprotective effect through suppressing oxidative stress.

Inflammation is one of the common causes for nerve damage, as a precipitating factor of POCD [25]. The pathogenesis of POCD remains unclear yet, with the neuroinflammatory mechanism as one of the most important theories currently. Therefore, it is necessary to study various inflammatory factors causing inflammation. The tissue damage caused by surgical trauma leads to massive release of various inflammatory factors, among which TNF-α, IL-1β, and IL-6 are the most representative. The elevation of their concentration indicates decline in cognitive function. TNF-α is an important inflammatory mediator in immune, inflammatory response, and neuroendocrine regulation, which plays critical roles in many links of life activities, including sleeping, eating, and other autonomous activities. IL-1β is an inflammatory factor with a wide range of biological activities, which can directly or indirectly pass through the blood-brain barrier. In this study, the correlation between the levels of inflammatory factors and cognitive dysfunction was explored, and the levels of TNF-α, IL-1β, and IL-6 in the hippocampus in SD + DEX group were lower than those in SD group, being consistent with previous literatures.

Zhou *et al*. found that the Rac1/AKT signaling pathway played an important regulatory role in inflammatory response [26]. NF-κB has attracted widespread attention because of its key role in regulating inflammation [27]. To confirm that the therapeutic effect of DEX on SD is related to the Rac1/AKT signaling pathway, the changes in protein expressions of Rac1/AKT/NF-κB and the entry of NF-κB into the nucleus in the hippocampus were detected in this study. The protein expressions of Rac1/AKT/NF-κB obviously rose in the hippocampus, and the proportion of NF-κB entering the nucleus increased in SD group. After DEX treatment, the protein expressions of Rac1/AKT/NF-κB and proportion of NF-κB entering the nucleus markedly declined, indicating that DEX may exert a therapeutic effect on SD via the Rac1/AKT/NF-κB signaling pathway.

## Conclusions

In conclusion, DEX may relieve oxidative stress damage in the hippocampus, neuronal damage, and inflammatory response in the hippocampal CA1 region of SD rats via the Rac1/AKT/NF-κB pathway, thereby improving the cognitive dysfunction.

## Highlights

- DEX alleviates hippocampal inflammation and improves cognitive function of SD rats.
- DEX relieves oxidative stress of hippocampal neurons and restores damaged cells.
- The ability of DEX may be related to the Rac1/AKT/NF-κB pathway.
